# Status and associated factors of food and nutrition literacy among young adults aged 15–44 years in Shenzhen City, China

**DOI:** 10.3389/fpubh.2023.1329241

**Published:** 2024-01-10

**Authors:** Li Zhixue, Xu Ying, Liu Zheng, Ma Yan, Guo Yanfang, Wang Dewang, Yu Weijun, Zhao Rencheng, Yuan Qing, Xu Meihong

**Affiliations:** ^1^Department of Chronic Disease Prevention and Control, Shenzhen Baoan Center for Chronic Diseases Control, Shenzhen, China; ^2^Department of Nutrition and Food Hygiene, School of Public Health, Peking University, Beijing, China

**Keywords:** China, Shenzhen, food and nutrition literacy, young adults, associated factors

## Abstract

**Background:**

Food and nutrition literacy (FNL) plays an important role in young adults’ dietary habits and nutrition. This study aimed to investigate FNL status and its associated factors among young adults aged 15–44 years in Shenzhen.

**Methods:**

A cross-sectional survey of 5,390 participants was conducted in June 2021. FNL was measured using the Food and Nutrition Literacy Questionnaire for Chinese Adults (FNLQ). A generalized linear model was employed to analyze the factors associated with FNL.

**Results:**

The median FNL score (total score = 100) was 68.00, which was below the adequate level of 80. FNL was divided into the two different domains of knowledge and skills, with significantly different scoring rate of 85.30 and 67.77%, respectively. The overall proportion of respondents with adequate FNL was 19.52%. The FNL score was significantly higher among the participants who were female (*β* = 2.665; 95% confidence interval [CI]: 2.031–3.299) and with higher education levels (*β* ranging from 5.632 [CI: 3.845–7.419] to 10.756 [CI: 8.973–12.538]), healthcare-related work experience (*β* = 4.197; CI: 3.557–4.837) and a higher economic status (*β* ranging from 0.753 [CI: 0.079–1.426] to 6.217 [CI: 5.208–7.227]). Those who were divorced or with an unknown marital status (*β* = −8.438; CI: −9.701, −7.175), abnormal body mass index (thin [*β* = −2.115; CI: −3.063, −1.166], overweight [*β* = −1.427; CI: −2.254, −0.600]), and suffering from chronic diseases (single disease [*β* = −3.483; CI: −4.485, −2.480], multimorbidity [*β* = −5.119; CI: −5.912, −4.327]) had significantly lower FNL scores.

**Conclusion:**

Generally, the level of FNL among young adults in Shenzhen, China, was relatively low. Thus, nutrition education programs targeted at promoting improved FNL status call for additional emphasis, especially in subgroups with lower scores.

## 1 Introduction

Chinese dietary patterns and diet-related behaviors have undergone significant transitions in the past few decades, with recent changes trending toward diversification and modernization (1, 2). The problem of malnutrition, which mainly manifests as the coexistence of insufficient and unbalanced nutrition intake, has thus become more prominent. Consequently, nutrition-related chronic diseases have been susceptible to rapid growth (3). Nutritional deficiencies was estimated to affect 8.31% of Chinese. At the same time, 34.8% of Chinese adults were overweight and 14.1% were obese (4). Food and nutrition literacy (FNL) is defined as an individual’s ability to obtain, understand, and process food and nutrition information and apply nutrition knowledge (5). It is regarded as a specific form of health literacy (6). FNL affects dietary behavior and nutrition intake, which leads to nutritional status disparities and further health impacts (7). Recent studies have suggested that malnutrition is closely related to FNL and poor dietary behavior (8). Specifically, individuals with low FNL are at a high risk of diet-related chronic diseases, such as diabetes/hyperglycemia, hypertension, coronary heart disease and stroke (9). It is worth noting that both FNL and dietary behavior can be improved by nutrition education and management (8). Thus, improving FNL has been regarded as an effective strategy to promote nutritional status and health.

In 2020, the frequency of adequate health literacy among the Chinese population increased from 6.48%, as recorded in 2008, to 23.15%, indicating a great improvement over the past 10 years (10). However, the current evaluation and monitoring system for health literacy in China is unable to evaluate the content of FNL. These existing measures do not accurately, objectively, or comprehensively reflect the status of FNL (11). At present, an established set of FNL assessment tools have been widely used among the general population outside of China, such as the Nutrition Literacy Scale (NLS) (12), the Nutrition Literacy Assessment Instrument (13), the Evaluation Instrument of Nutrition Literacy in Adults (14), and the Japan Nutrition Literacy Scale (15). However, due to significant differences in dietary habits, socioeconomic development, and cultural backgrounds, the above FNL assessment tools are not suitable for China’s distinctive national conditions. Accordingly, some studies have used self-designed questionnaires to explore the status of nutrition knowledge and practice, as well as its correlation with chronic diseases, including diabetes/hyperglycemia, hypertension, and dyslipidemia (9, 16). However, there is still a lack of recognized evaluation methods and tools for assessing FNL for Chinese adults. In this regard, the FNLQ was established as having good validity and reliability (Cronbach’s α = 0.893, χ^2^/DF = 4.750, root mean square error of approximation [RMSEA] = 0.048, goodness-of-fit index [GFI] = 0.891 and adjusted goodness-of-fit index [AGFI] = 0.876). Thus, the FNLQ could be considered a promising scale for assessing the FNL of Chinese adults (17).

Shenzhen is one of the youngest and fastest growing cities in China. Unprecedented social and economic development in Shenzhen has led to significant changes in dietary patterns, which have largely contributed to the high prevalence of nutrition-related chronic diseases (e.g., excess body weight and dyslipidemia) (18, 19). Furthermore, the FNL status of young adults and its correlates remain to be examined in Shenzhen. Therefore, this study aimed to investigate and analyze the status of FNL and its associated factors using the FNLQ among young adults aged 15–44 years in Shenzhen. The findings are expected to assist public health authorities in developing strategies for FNL promotion based on sound references and theoretical foundations.

## 2 Materials and methods

### 2.1 Data collection

In June 2021, a stratified sampling approach was employed to recruit participants from each district of Shenzhen. The number of sample was determined according to the population’s proportion of gender and age. The study participants were citizens aged 15–44 living in Shenzhen, China, who were willing to participate in the survey. Participants were asked to complete an online survey anonymously and independently. All participants were informed about the study, and consent was obtained before completing the survey. Cross-checking was conducted to eliminate invalid questionnaires due to a lack of information or implausible answers. The study was approved by the Human Research Ethics Committee of the Shenzhen Baoan Center for Chronic Diseases Control (approval number: SZBACCDC-2021013).

### 2.2 Measures

The questionnaire mainly included two sections: (1) the first section asked for basic information about the residents, namely their gender, nationality, age, educational level, marital status, health-related work experience, economic status, body height and weight, and chronic disease history; and (2) the second section comprised a 20-component survey of FNLQ, which consisted of two domains (knowledge and skills) and four dimensions (food and nutrition knowledge) [component 1–4], selecting food [component 5–10], preparing food [component 11–13], eating [component 14–20] (see Table 1). The questionnaire was developed and validated for Chinese adults, as reported elsewhere (17).

**Table 1:** The scores of the core items of food and nutrition literacy.

Domain	Dimension	No.	Components	Number of questions	Total score	M (P_25_–P_75_)	Scoring rate (%)	
Knowledge	Food and nutrition knowledge	1	Understanding that a healthy diet should be followed at every stage of life.	2	4	2.00 (1.00–2.00)	85.93
		2	Understanding that a rational diet is an important basis for maintaining health and avoiding disease.	2	4	2.00 (1.00–2.00)	85.57
		3	Knowing about food classification, sources, and main nutritional characteristics.	2	4	3.50 (2.00–4.00)	84.22
		4	Choosing a healthy diet and enjoy your food.	1	2	2.00 (1.00–2.00)	86.58
Skills	Selecting food	5	Making your own food, eating out less and sharing meals with family.	2	4	4.00 (2.00–4.00)	85.28
		6	Being able to choose safe and hygienic food stores and restaurants.	2	4	3.00 (1.00–5.00)	50.12
		7	Being able to judge food quality and to choose fresh and healthy food.	1	2	2.00 (0.00–2.00)	57.03
		8	Being able to read and understand food nutrition labels.	3	6	3.00 (1.00–5.00)	50.50
		9	Paying attention to nutrition and health information, identifying, and applying the right information.	3	6	3.50 (1.00–6.00)	59.16
		10	Being able to choose healthy food and fortified food correctly.	3	6	4.00 (1.50–5.00)	58.25
	Preparing food	11	Being able to estimate food portion size.	2	4	3.00 (1.00–4.00)	67.60
		12	Being able to match food rationally.	1	2	2.00 (0.00–2.00)	52.02
		13	Being able to store, prepare, process, and cook food in an appropriate manner.	8	16	9.50 (5.50–14.00)	59.65
	Eating	14	Eating regular meals and having a good breakfast.	1	2	2.00 (0.50–2.00)	82.55
		15	Eating a variety of foods, mainly grains, eating more fruits and vegetables, and drinking plenty of water.	5	10	8.00 (4.5–10.00)	76.91
		16	Eating appropriate amount of fish, poultry, eggs, lean meat, and adequate milk and beans.	2	4	3.00 (1.50–4.00)	74.13
		17	Eating less salt and less oil, controlling sugar, and limiting alcohol.	3	6	3.50 (2.00–6.00)	59.20
		18	Preparing meals on demand, eating in a civilized manner, and eliminating waste.	2	4	4.00 (2.00–4.00)	86.21
		19	Respecting different food cultures and paying attention to table manners.	1	2	2.00 (1.00–2.00)	85.95
		20	Balance eating and movement, measure and evaluate your weight regularly.	5	10	6.50 (4.00–9.50)	66.71
Total				50	100	68.00 (44.50–87.00)	67.15

The FNLQ included 50 questions, with a total possible score of 100 points. The questions included 5-point Likert-type questions (e.g., for a statement such as, “Good dietary patterns are the foundation of adequate nutrition,” respondents could indicate that they strongly disagree, disagree, do not know, agree, or strongly agree) and multiple-choice questions with only one right answer (e.g., “What is the approximate weight of a ping-pong-ball-sized egg?”). The higher the score, the higher the FNL level of the respondents. An FNL score higher than 80 was considered to indicate an adequate level of FNL. The scores of the different domains and dimensions were converted into scoring rates for comparison as follows:


$$\begin{array}{l} \mathrm{Domain}\;\mathrm{scoring}\;\mathrm{rate}=\sum \mathrm{score}\;\mathrm{of}\;\mathrm{the}\;\mathrm{domain}\;for\;\mathrm{every}\;\mathrm{respondent}/\\ \;\left(\begin{array}{l} \mathrm{full}\;\mathrm{score}\;\mathrm{of}\;\mathrm{the}\;{\mathrm{domain}}^{*}\;\mathrm{the}\;\\ \mathrm{number}\;\mathrm{of}\;\mathrm{respondents} \end{array}\right) \end{array}$$

$$\begin{array}{l} \mathrm{Dimension}\;\mathrm{scoring}\;\mathrm{rate}=\sum \mathrm{score}\;\mathrm{of}\;\mathrm{the}\;\mathrm{dimension}\;for\;\mathrm{every}\;\\ \;\mathrm{respondent}/\left(\begin{array}{l} \mathrm{full}\;\mathrm{score}\;\mathrm{of}\;\mathrm{the}\;\\ {\mathrm{dimension}}^{*}\;\mathrm{the}\;\mathrm{number}\;\\ \mathrm{of}\;\mathrm{respondents} \end{array}\right) \end{array}$$

### 2.3 Statistical analysis

After checking all the questionnaires and excluding the invalid ones, all data were imported into the Statistical Package for the Social Sciences 24.0 software program (SPSS, Inc., Chicago, Illinois, USA) for statistical analysis. As the FNL score did not exhibit a normal distribution, it was represented as median and percentile 25 and 75 (M[P_25_–P_75_]). The Wilcoxon rank sum test and Kruskal–Wallis test were applied to compare the median differences. Categorical variables were reported by frequencies and percentages of distribution. A chi-square test was used to examine whether the distribution of categorical variables between the groups was significantly different, and Spearman’s correlation coefficient was calculated to examine the bivariate association between the different domains of FNL. Generalized linear and logistic linear regression analyses were used to explore the factors related to FNL. The statistical significance level was set at *p* < 0.05.

## 3 Results

### 3.1 Demographic characteristics of the study participants

This investigation included 5,390 valid questionnaires, of which 53.19% (2867) and 46.81% (2523) were completed by males and females, respectively, with an average age of 33.68 ± 4.60 years. The majority of the respondents was 35–44 years old (57.24%; *n* = 3,085), and they were mainly Han Chinese (99.54%; *n* = 5,365). Educational level was mainly junior college/undergraduate and above (64.25%; *n* = 3,463), followed by junior high school/senior high school or secondary specialized school (32.19%; *n* = 1,735). Most of the respondents were married (76.81%, *n* = 4,140). A total of 47.83% (*n* = 2,578) had health-related work experience. According to the BMI cutoff in the dietary guidelines for Chinese residents (2016) (20), 12.76% (*n* = 672) and 17.38% (*n* = 915) of the respondents were thin (BMI = 23.9–27.9) and overweight (BMI > 24), respectively (see Table 2 for additional information).

**Table 2:** Demographic characteristics of study participants (*N* = 5,390).

Demographic characteristics		*n*	%
Gender	Male	2,867	53.19
	Female	2,523	46.81
Age group (year)	15–24	333	6.18
	25–34	1972	36.59
	35–44	3,085	57.24
Educational level	Primary school and below	192	3.56
	Junior high school/senior high school or secondary specialized school	1735	32.19
	Junior college/undergraduate or above	3,463	64.25
Marital status	Unmarried	848	15.73
	Married	4,140	76.81
	Others	402	7.46
Ethnicity	Han	5,365	99.54
	Minority	25	0.46
Healthcare-related work experience	Yes	2,578	47.83
	No	2,812	52.17
Family income *per capita* (CNY/month)	≤ 13,000	2,846	52.80
	13,000 ~ 24,000	1906	35.36
	≥ 24,000	638	11.84
BMI group	Thin	672	12.76
	Normal	3,678	69.86
	Overweight	915	17.38
Chronic diseases	None	3,509	65.10
	Single disease	589	10.93
	Multimorbidity	1,292	23.97

Chronic diseases (suffering from two diseases at the same time was judged as multimorbidity, including dyslipidemia, diabetes or elevated blood sugar, hypertension, cancer and other malignant tumors, chronic lung diseases like bronchitis, emphysema, pulmonary heart disease, liver diseases, heart disease, stroke, kidney disease, stomach disease or digestive system disease, emotional or other mental problems, etc.).

### 3.2 Food and nutrition literacy status

**Figure 1:**
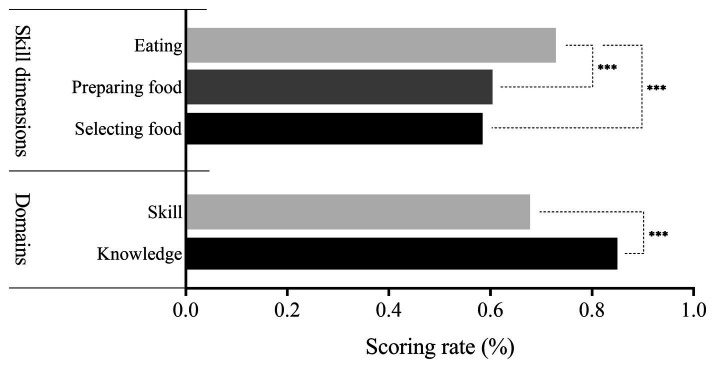
Scoring rate among different dimensions of FNL. *p < 0.05; **p < 0.01; **p < 0.001.

As shown in Table 1, the median FNL score was 68.00 (44.50, 87.00). The overall proportion of respondents with adequate FNL was 19.52% (1,052/5,390). Among the FNL domain, the scoring rate for knowledge was 85.30%, which was significantly higher than that of skill (67.77%). For the skill dimensions, the scoring rate for selecting food was the lowest (58.78%), followed by preparing food (60.40%) and then eating (72.89%) (*p* < 0.05) (Figure 1).

In terms of selecting food, the scoring rate for the components of “being able to read and understand food nutrition labels” and “being able to choose safe and hygienic food stores and restaurants” were the lowest, with scoring rates of 50.50 and 50.12%, respectively. In terms of preparing food, the scoring rate for the component of “being able to match food rationally” was the lowest at 52.02%. In terms of eating, the scoring rate for the components of “eating less salt and less oil, controlling sugar, and limiting alcohol” and “balance eating and movement; measure and evaluate your weight regularly” were the lowest, with scoring rates of 59.20 and 66.71%, respectively (Table 1).

As shown in Table 3, a significant correlation was found between total FNL and knowledge (*r* = 0.698) and skills (*r* = 0.996). The correlation coefficients between total FNL and each dimension ranged from 0.698 to 0.891 (*p* < 0.05). Higher skill scores were significantly correlated with higher knowledge scores (*r* = 0.642). The correlation coefficients between each dimension ranged from 0.373 to 0.903 (*p* < 0.05).

**Table 3:** Spearman correlation coefficients among the domains and dimensions of FNL (*N* = 5,390).

Variables		Total FNL and its domains			Skill dimensions		
		Total FNL	Knowledge	Skills	Selecting food	Preparing food	Eating
Total FNL and its domains	Total FNL	–	0.698^**^	0.996^**^	0.891^**^	0.840^**^	0.747^**^
	Knowledge	0.698^**^	–	0.642^**^	0.484^**^	0.422^**^	0.698^**^
	Skill	0.996^**^	0.642^**^	–	0.903^**^	0.855^**^	0.729^**^
Skill dimensions	Selecting food	0.891^**^	0.484^**^	0.903^**^	–	0.783^**^	0.456^**^
	Preparing food	0.840^**^	0.422^**^	0.855^**^	0.783^**^	–	0.373^**^
	Eating	0.747^**^	0.698^**^	0.729^**^	0.456^**^	0.373^**^	–

–, no data; *p < 0.05; **p < 0.01; ***p < 0.001.

### 3.3 Distribution of food and nutrition literacy

**Figure 2:**
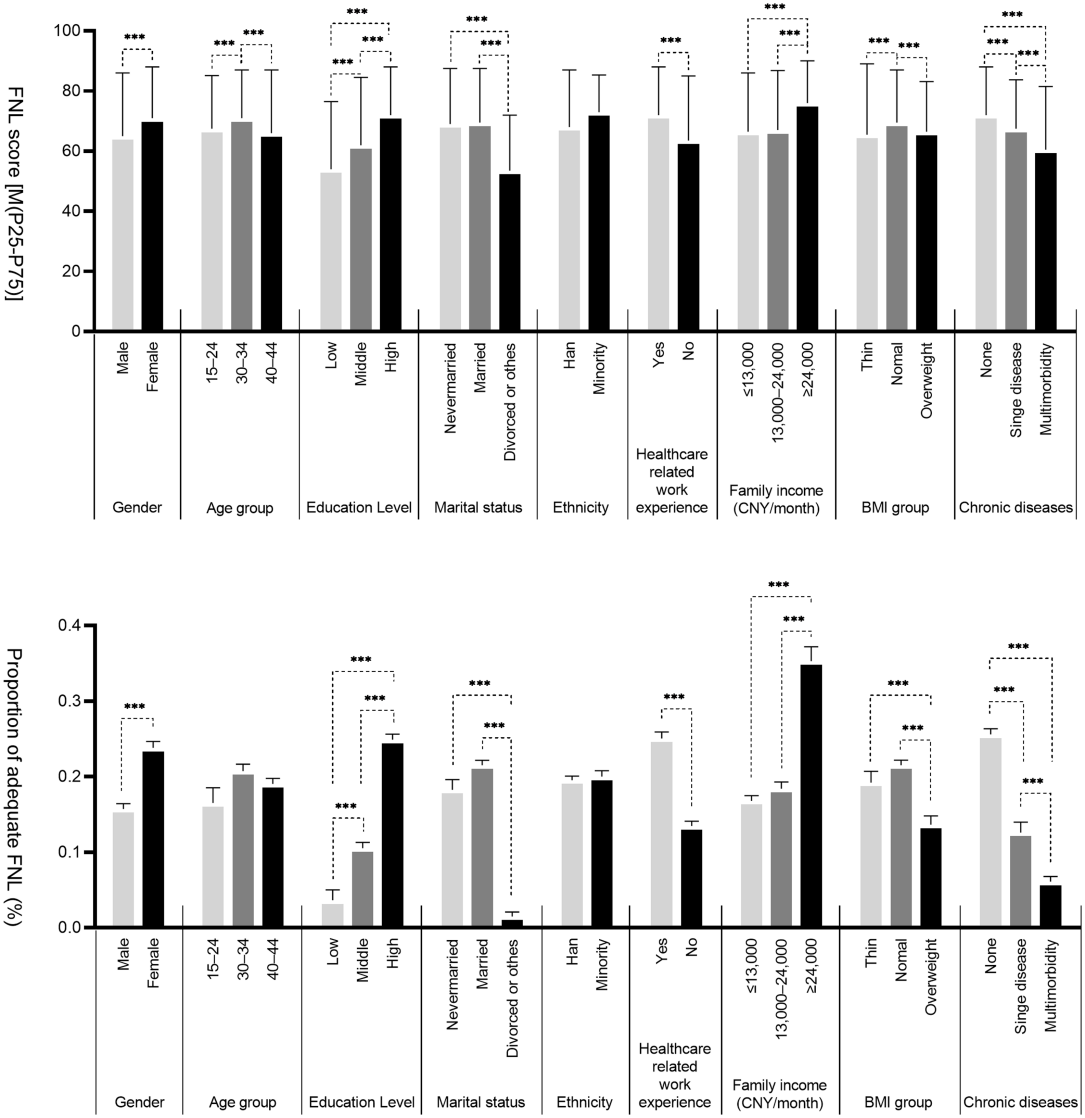
Distribution of FNL in young adults aged 15-44 in Shenzhen. Education level (Low: Primary school and below, Middle: Junior high school/senior high school or secondary specialized school, High: Junior college/undergraduate and above); Family income (Monthly income per capita = total family income/total number of family members, CNY: Chinese yuan); Chronic disease (Suffering from the two diseases at the same time were judged as multimorbidity, including dyslipidemia, diabetes or elevated blood sugar, hypertension, cancer and other malignant tumors, chronic lung diseases such as bronchitis, emphysema, pulmonary heart disease, liver diseases, heart disease, stroke, kidney disease, stomach disease or digestive system disease, emotional, and mental problems, etc.); *p < 0.05; **p < 0.01; ***p < 0.001.

As presented in Figure 2, both the FNL scores and the proportion of participants with adequate FNL were significantly higher in participants with the following characteristics: female (versus male), nonsmokers (versus smokers), higher level of education, married or never married, (versus divorced and other marital status), healthcare-related work experience (versus no such experience), higher family income, normal BMI (versus abnormal BMI < 18.5 or ≥ 24.0), and no chronic disease (versus suffering from chronic diseases) (*p* < 0.05). The FNL score was also significantly higher in participants aged 25–34 years compared to those aged 15–24 years and 35–44 years.

### 3.4 Multivariate analysis of food and nutrition literacy

The multivariate analysis showed that the FNL scores of females were higher than those of males (*β* = 2.665, CI: 2.031–3.299). A higher educational level (*β* ranging from 5.632 [CI: 3.845–7.419] to 10.756 [CI: 8.973–12.538]) and higher family income (*β* ranging from 0.753 [CI: 0.079–1.426] to 6.217 [CI: 5.208–7.227]) were also associated with an increased FNL score. The FNL scores of those who had never married (*β* = −1.309, CI: −2.255, −0.364) and those who were divorced or had another unknown marital status (*β* = −8.438; CI: −9.701, −7.175) were lower than those who were married. Health-related work experience was correlated with a higher FNL score compared to those without such experience (*β* = 4.197, CI: 3.557–4.837). The highest FNL score was also shown in the normal BMI group versus the abnormal BMI group (thin [*β* = −2.115; CI: −3.063, −1.166], overweight [*β* = −1.427; CI: −2.254, −0.600]). Suffering from a chronic disease decreased the FNL score compared to those who were healthy (singe disease [*β* = −3.483; CI: −4.485, −2.480], multimorbidity [*β* = −5.119; CI: −5.912, −4.327]).

Similarly, adequate FNL was more likely to be associated with being female, well-educated, having a stable marital status (never married or married), having healthcare-related work experience, having a higher family income, having a normal BMI, and not suffering from a chronic disease. Otherwise, participants with the lower age was were less likely to have adequate FNL (Table 4).

**Table 4:** Multivariate analysis of FNL among young adults aged 15–44 in Shenzhen (*N* = 5,390).

Demographic characteristics	Total FNL score^a^			Adequate FNL^b^		
	*β* *(95% CI)*	*Wald* *χ*^2^	*p* *value*	*OR (95% CI)*	*Wald* *χ*^2^	*p* *value*
Gender (Male)	Reference			Reference		
Female	2.665 (2.031–3.299)	67.841	< 0.001	1.475 (1.270–1.714)	25.946	< 0.001
Age group (Year) (15–24)	Reference			Reference		
25–34	1.541 (0.145–2.937)	4.680	0.031	0.614 (0.431–0.875)	7.272	0.007
35–44	1.401 (−0.024–2.826)	3.713	0.054	0.738 (0.630–0.864)	14.263	< 0.001
Educational level (Primary school and below)	Reference			Reference		
Junior high school/senior high school or secondary specialized school	5.632 (3.845–7.419)	38.169	< 0.001	2.218 (1.004–4.900)	3.880	0.049
Junior college/undergraduate and above	10.756 (8.973–12.538)	139.892	< 0.001	4.465 (2.041–9.769)	14.036	< 0.001
Marital status (Married)	Reference			Reference		
Never married	−1.309 (−2.255– −0.364)	7.364	0.007	0.758 (0.606–0.949)	5.833	0.016
Divorced or others	−8.438 (−9.701– −7.175)	171.410	< 0.001	0.106 (0.047–0.242)	28.560	< 0.001
Ethnicity (Minority)	Reference			Reference		
Han	1.112 (−3.344–5.567)	0.239	0.625	0.823 (0.292–2.316)	0.136	0.712
Healthcare-related work experience (No)	Reference			Reference		
Yes	4.197 (3.557–4.837)	165.291	< 0.001	1.689 (1.449–1.969)	45.033	< 0.001
Family income (CNY/month) (≤ 13,000)	Reference			Reference		
13,000–24,000	0.753 (0.079–1.426)	4.799	0.028	1.099 (0.934–1.294)	1.287	0.257
≥ 24,000	6.217 (5.208–7.227)	145.648	< 0.001	2.440 (1.981–3.006)	70.455	< 0.001
BMI group (Normal)	Reference			Reference		
Thin	−2.115 (−3.063–-1.166)	19.084	<0.001	0.836 (0.665–1.051)	2.346	0.126
Overweight	−1.427 (−2.254, −0.600)	11.444	< 0.001	0.645 (0.519–0.801)	15.776	< 0.001
Chronic diseases (None)	Reference			Reference		
Single disease	−3.483 (−4.485, −2.480)	46.374	< 0.001	0.448 (0.344–0.583)	35.431	< 0.001
Multimorbidity	−5.119 (−5.912, −4.327)	160.163	< 0.001	0.277 (0.216–0.357)	99.919	< 0.001

^a^Multivariate linear regression model; ^b^Logistic regression model; CNY: Chinese yuan: Chronic diseases (suffering from two diseases at the same time was judged as multimorbidity, including dyslipidemia, diabetes or elevated blood sugar, hypertension, cancer and other malignant tumors, chronic lung diseases like bronchitis, emphysema, pulmonary heart disease, liver diseases, heart disease, stroke, kidney disease, stomach disease or digestive system disease, emotional or other mental problems, etc.).

## 4 Discussion

FNL is an important content indicator of health literacy (11). It is also an essential factor in improving nutrition status and in preventing and controlling nutrition-related chronic diseases (21–24). In this study, the FNLQ was used to analyze the FNL of young adults aged 15–44 and its related factors in Shenzhen, China. The results showed that the median FNL score was only 68.00, which is below the minimum adequate level of 80. Accordingly, the probability of obtaining an adequate FNL score was only 19.52%, which indicates that the FNL status of young adults in this region needs further improvement. In addition, gender, age, educational level, marital status, healthcare-related work experience, family income, BMI classification, and health status were evaluated as factors related to FNL.

FNL has been divided into functional FNL, interactive FNL, and critical FNL (6). People are expected not only to master nutrition knowledge and skills, but also to exhibit the ability to make good decisions and address more complex nutrition issues as they arise (6, 25, 26). Therefore, FNL is regarded as an integral part of nutrition education programs and is crucial for health promotion (27). Our results revealed a significantly moderate correlation between knowledge score and total FNL score (*r* = 0.698) and skill score (*r* = 0.642). The necessity of food and nutrition knowledge as a prerequisite for dietary changes (28), although insufficient, calls for more emphasis on nutrition educational programs. However, the scoring rate for skills were lower than that for knowledge. Thus, improving skills might be a key strategy for upgrading the status of FNL. It is worth noting that a significant but poor correlation was found between preparing food and eating (*r* = 0.373) among the skill dimensions. Comprehensive intervention measures should be taken to improve each dimension of skills more evenly.

In the dimension of selecting food, the scoring rate for the components of “being able to read and understand food nutrition labels” and “being able to choose safe and hygienic food stores and restaurants” were the lowest. Food labels include accurate information about expiration dates, ingredient lists, and the nutritional value of foods (29). They have been designed to help Chinese consumers understand food and nutrition information, which is expected to enable them to choose healthy foods. However, food labels are rarely used by Chinese consumers when shopping for food (30). The results of a review study showed that Chinese commercial and residential food handlers had insufficient food safety knowledge, especially in the areas of foodborne pathogens and safe food-handling practices. It is thus necessary to improve the public’s capacity to exercise food safety (31). Thus, future FNL-promoting programs should pay attention to skills assessment, which should focus on food and nutrition information acquisition, decision making, and safe practices.

In addition to making the appropriate food selections, preparing food is also an important part of balanced nutrient intake. Food categories, eating frequency, and cooking methods are gradually increasing and diversifying in China (1, 32). To improve diet-related behaviors, the 2022 Dietary Guidelines for Chinese residents have proposed reasonable food collocation which mainly refers to: following a healthy dietary pattern, and mastering the simple principles of food diversity, collocations between the staple and subsidiary food, coarse and fine grain, animal and vegetarian food, food with various colors, etc. (33). In terms of preparing food, the scores for the component “being able to match food rationally” were the lowest. This indicates that awareness of food collocation is poor among young adults in Shenzhen. According to the literature, dietary collocation and the daily intake of foods can notably be improved after a nutrition education intervention (34). Thus, we need to pay greater attention to addressing these weaker links in the preparing food dimension.

In another study, FNL was found to predict adherence to healthy/unhealthy dietary patterns (35). In the dimension of eating, the scores for the components of “eating less salt and less oil, controlling sugar, and limiting alcohol” and “balance eating and movement; measure and evaluate your weight regularly” were the lowest. According to the literature, the macronutrient composition of the diets consumed by Chinese adults has shifted toward fats, and sodium intake remains high (1, 32). Moreover, the intake of foods high in added sugar has increased (32). Under the increasing prevalence of alcohol use, low physical activity, and high BMIs, the disease burden attributed to these factors in China showed an overall upward trend from 1990 to 2019 (36). Therefore, further promoting the “China Healthy Lifestyle for All” project (where healthy lifestyles include salt, oil, and sugar reduction and healthy weight maintenance) is an important strategy to improve the FNL of young adults (37).

The results of our study showed that females had higher FNL levels than males. Chinese females are often responsible for taking care of their families and housework, including recipe and food selection and preparation (15, 38). Thus, they have more opportunities to learn, master, and use nutrition-related information and services more actively. Similarly, married people showed higher FNL scores, indicating that they may apply dietary knowledge into practice due to their own or their family’s nutrition and health (39). At present, the average age of the permanent population in Shenzhen is less than 35 years, and youth may be more included to neglect nutrition and health knowledge due to factors such as a fast-paced work environment and the lack of nutrition education curricula. The young age group in our study had low FNL scores, which highlights the need for special attention to be directed toward this group. Higher educational level and economic status were also positively associated with FNL. Consistent results have been reported in several other studies (15, 40, 41). Furthermore, a review of the available literature indicates that abnormal BMI and suffering from a chronic disease have been associated with a low probability of achieving a high level of FNL (9, 17, 42), which is also consistent with our findings. Thus, these factors should be included in key publicity and education initiatives.

To the best of our knowledge, this is the first study to assess the FNL status of young adults in Shenzhen using a valid multidimensional tool. However, several limitations need to be noted. First, its cross-sectional design makes it impossible to interpret the direction of associations. Moreover, our results are based on an online investigation of the respondents’ self-reported measures. Therefore, memory and reporting biases were possible. However, they may have been partly overcome by the large sample size, as well as by the anonymity and confidentiality of the data. Finally, this study was conducted among young adults aged 15–44 in Shenzhen, and the participants showed a relatively high education level. Therefore, its results may not be generalized to other age groups or different populations.

In conclusion, the present study showed that young adults in Shenzhen have relatively low FNL, especially in the food and nutrition-related skill dimensions. Among the possible associated factors examined, age, gender, educational level, health-related work experience, family income, marital status, BMI group, and health status were all significantly correlated with FNL. Further studies are recommended to identify other possible factors related to FNL. For example, FNL may be affected by social support levels that were not assessed in the present study (15). Nutrition education programs with content pertaining to food and nutrition knowledge, skills, and behaviors call for additional development emphasis. The findings have also highlighted the need for future studies focusing on FNL-promoting interventions for young adults in Shenzhen.

## Author contributions

LiZ: Data curation, Formal analysis, Investigation, Methodology, Software, Visualization, Writing – original draft, Writing – review & editing, Funding acquisition. XY: Conceptualization, Resources, Validation, Writing – review & editing. LiuZ: Resources, Validation, Writing – review & editing. MY: Investigation, Methodology, Writing – review & editing. GY: Investigation, Methodology, Writing – review & editing. WD: Investigation, Methodology, Writing – review & editing. YW: Investigation, Methodology, Writing – review & editing. ZR: Investigation, Methodology, Writing – review & editing. YQ: Project administration, Resources, Supervision, Writing – review & editing. XM: Conceptualization, Project administration, Resources, Supervision, Validation, Writing – review & editing.
